# Phenotype-Based Screening of Synthetic Cannabinoids in a Dravet Syndrome Zebrafish Model

**DOI:** 10.3389/fphar.2020.00464

**Published:** 2020-04-24

**Authors:** Aliesha Griffin, Mana Anvar, Kyla Hamling, Scott C. Baraban

**Affiliations:** Epilepsy Research Laboratory and Weill Institute for Neuroscience, Department of Neurological Surgery, University of California San Francisco, San Francisco, CA, United States

**Keywords:** cannabinoid, epilepsy, locomotion, screen, seizure, zebrafish

## Abstract

Dravet syndrome is a catastrophic epilepsy of childhood, characterized by cognitive impairment, severe seizures, and increased risk for sudden unexplained death in epilepsy (SUDEP). Although refractory to conventional antiepileptic drugs, emerging preclinical and clinical evidence suggests that modulation of the endocannabinoid system could be therapeutic in these patients. Preclinical research on this topic is limited as cannabis, delta-9-tetrahydrocannabinol (THC) and cannabidiol (CBD), are designated by United States Drug Enforcement Agency (DEA) as illegal substances. In this study, we used a validated zebrafish model of Dravet syndrome, scn1lab homozygous mutants, to screen for anti-seizure activity in a commercially available library containing 370 synthetic cannabinoid (SC) compounds. SCs are intended for experimental use and not restricted by DEA designations. Primary phenotype-based screening was performed using a locomotion-based assay in 96-well plates, and a secondary local field potential recording assay was then used to confirm suppression of electrographic epileptiform events. Identified SCs with anti-seizure activity, in both assays, included five SCs structurally classified as indole-based cannabinoids JWH 018 N-(5-chloropentyl) analog, JWH 018 N-(2-methylbutyl) isomer, 5-fluoro PB-22 5-hydroxyisoquinoline isomer, 5-fluoro ADBICA, and AB-FUBINACA 3-fluorobenzyl isomer. Our approach demonstrates that two-stage phenotype-based screening in a zebrafish model of Dravet syndrome successfully identifies SCs with anti-seizure activity.

## Introduction

In patients classified with catastrophic epilepsies in childhood, effective seizure control using conventional antiepileptic drugs (AEDs) can be a significant problem. Early developmental AED exposure can also be associated with undesirable side effects in these children. In the search for new treatments, there is growing interest in drugs modulating the endogenous cannabinoid system. The endocannabinoid system, comprised of two G-protein coupled receptors (CB1 and CB2), may play a role in regulating seizure activity (Wallace et al., 2002; Lutz, 2004; Deshpande et al., 2007). Recent data suggests potential anticonvulsant activity for synthetic cannabinoids (SCs), phytocannabinoids, and *Cannabis sativa* (cannabidiol) e.g., drugs targeting the endocannabinoid system (Rosenberg et al., 2017; Smolyakova et al., 2020). Unfortunately, careful preclinical examination of cannabis and related extracts is difficult as they are designated illegal schedule I compounds. Potent SCs, representing compounds engineered to bind cannabinoid receptors with high affinity, offer an alternative. To date, these compounds have been examined most extensively in animal models of acute seizures, with mixed results. In the pentylenetetrazole (PTZ) model of generalized seizures, WIN 55,212-2 (a mixed CB1/CB2 receptor agonist), exerts pro- and anticonvulsant effects whereas arachidonyl-2'-chloroethylamide (a CB1 receptor agonist) was shown to decrease acute PTZ seizure thresholds, increase acute PTZ seizure thresholds or have no effect at all (Gholizadeh et al., 2007; Shafaroodi et al., 2008; Naderi et al., 2011; Andres-Mach et al., 2012; Vilela et al., 2013). In the maximal dentate activation model of limbic seizures or limbic kindling models, WIN 55,212-2 reduced seizure thresholds (Wallace et al., 2001; Colangeli et al., 2017), delayed epileptogenesis (Di Maio et al., 2015), or failed to provide any seizure protection (Luszczki et al., 2011). Recent testing completed at the Epilepsy Therapy Screening Program reported anti-seizure properties for cannabidiol (CBD) in mouse 6 Hz 44 mA and mouse/rat maximal electroshock seizure assays (Klein et al., 2017). Although even less is known about experimental models of childhood epilepsies, a recent study by Huizenga et al., 2017 examined seven cannabinoid receptor agonists in young postnatal rat models of PTZ- hypoxia- or methyl-6,7-dimethoxy-4-ethyl-beta-carboline-3-carboxylate (DMCM)-evoked seizures, only WIN 55,212-2 exhibited anti-seizure activity in all three models.

Interestingly, Dravet syndrome—a genetic epilepsy associated, in most cases, with a loss-of-function *de novo* mutation in the *SCN1A* voltage-gated sodium channel subunit (Gataullina and Dulac, 2017)—is one particular form of childhood epilepsy where CBD has shown anticonvulsant activity. Reductions in thermally-induced and spontaneous seizure activity were observed in *Scn1a^+/^* ^−^ mice at CBD concentrations above 100 mg/kg with amelioration of autistic-like behaviors seen at a lower concentration of 10 mg/kg (Kaplan et al., 2017). In the first open-label investigational trial of CBD in children with Dravet syndrome, Devinsky et al. (2016) reported a 37% median reduction in monthly seizure counts. Additional positive seizure reductions with CBD treatment (Porter and Jacobson, 2013; Crippa et al., 2016; McCoy et al., 2018), and a GW Pharmaceuticals sponsored open-label double-blinded study reported an adjusted 23% reduction in seizure frequency led to FDA approval for CBD (Epidiolex^®^) as a treatment for seizures associated with Dravet syndrome (Devinsky et al., 2017). Despite these advances, preclinical studies remain quite limited as laboratories using controlled substances (including research involving animals) are subject to extensive state and federal regulatory requirements (Samanta, 2019).

Here, we used an established *scn1* zebrafish mutant model of Dravet syndrome to screen synthetic compounds for those that exhibit anti-seizure properties. This commercially available library contains 370 parent compounds and positional isomers, analogs, or homologs designed for experimental, non-human, research purposes. The *scn1lab* mutant zebrafish line exhibits spontaneous seizures that can be easily monitored using acute behavioral and electrophysiological assays (Baraban et al., 2013; Hong et al., 2016; Griffin et al., 2017; Hunyadi et al., 2017). These mutants exhibit metabolic deficit, early fatality, sleep disturbances, and a pharmacological profile similar to Dravet syndrome patients (Schoonheim et al., 2010; Kumar et al., 2016; Grone et al., 2017). In addition to replicating key aspects of Dravet syndrome, *scn1lab* mutants were shown to be a useful model system for large-scale phenotype-based drug screening and experimental drugs identified in this model have shown efficacy in the clinic (Baraban et al., 2013; Dinday and Baraban, 2015; Griffin et al., 2017; Sourbron et al., 2017; Griffin et al., 2018). Here, we used *scn1lab* mutants to screen a SC library. We identified five compounds that exert significant anti-seizure activity during acute exposures. Structure-activity relationship analysis revealed these are indole-derived cannabinoids (Wiley et al., 1998) suggesting specific classes of SCs exert antiepileptic activity and providing further justification for using larval zebrafish models to identify novel therapeutic targets.

## Materials and Methods

### Zebrafish Maintenance

Zebrafish were maintained according to standard procedures. Ethical practices for the laboratory use of zebrafish followed guidelines approved by the Office of Ethics and Compliance branch of the Institutional Animal Care and Use Committee (IACUC approval #: AN171512-03) at the University of California, San Francisco. The zebrafish room was maintained on a light-dark cycle, with lights-on at 9:00 AM and lights-off at 11:00 PM. An automated feedback control unit was used to maintain aquarium water conditions in the following ranges: 29–30°C, pH 7.5–8.0, conductivity (EC) 690–710. Zebrafish embryos and larvae were raised in an incubator maintained at 28.5°C, on the same light-dark cycle as the fish facility. Water used for embryos and larvae was made by adding 0.03% Instant Ocean and 0.000002% methylene blue to reverse-osmosis distilled water. Embryos and larvae were raised in plastic petri dishes (90 mm diameter, 20 mm depth) and housing density was limited to approximately 50–60 per dish. The sex of embryos and larvae cannot be determined at these early stages.

### Drugs

**Figure 1:**
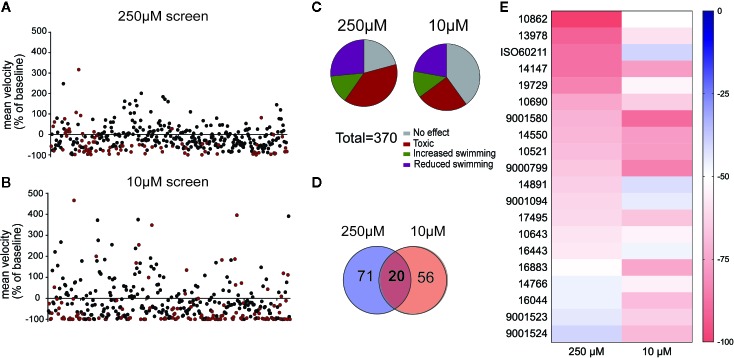
A library of synthetic cannabinoids (SCs) was screened for their ability to reduce the high velocity seizure-like swim behavior of 5 day old zebrafish larvae. Compounds were screened at **(A)** 250 µM and **(B)** 10 µM. Each data point represents the mean velocity change in swim behavior of six fish treated with an individual compound. The red data points represent compounds that failed to go into solution or identified as toxic after 90-min exposure. **(C)** Summary of compound effects after screening 370 SCs at 250 and 10 µM. The threshold for inhibition of seizure activity was determined as a reduction in mean swim velocity of ≥40%. **(D)** The total number of compounds identified as positive from the 250 and 10 µM library screen. **(E)** Heat map representing the 20 compounds which successfully reduced the mean swim velocity by >40% in the 250 and 10 µM screening.

**Figure 2:**
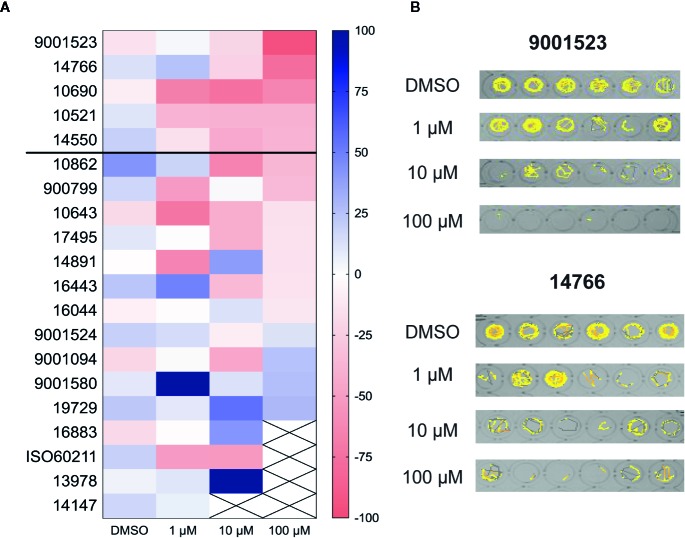
Behavioral screening of 20 compounds which were identified as positive from the library screens. **(A)** Heat map representing the change of mean swim velocity of the 20 hit compounds. The 20 SCs which were identified from the blind screens were retested at 1, 10, and 100 µM to confirm a dose response effect. Each box represents the percent change in mean velocity from a locomotion assay on at least 6 *scn1lab* larvae. Compounds marked with a cross were identified as toxic; threshold for positive hits shown by solid line. Scale at right represents the range of % change in mean velocity values. **(B)** Representative swim behavior traces obtained during a 10 min recording epoch for the top two compounds, #9001523 and #147666.

Compounds for drug screening were purchased from Cayman Chemicals and provided as 10 mM stock DMSO solutions (**Supplemental Table I**). Fresh drug solutions were prepared on each day of experimentation in 1 ml of E3 media. Final DMSO concentration of drug dilutions used for testing was ~1%. To facilitate large-scale primary screening (**Figure 1**), single drug concentrations (10 and 250 µM) were selected based on previously published high-throughput screens in larval zebrafish (Baraban et al., 2013; Robertson et al., 2014; Dinday and Baraban, 2015; Lin et al., 2018; Ibhazehiebo et al., 2018; Eimon et al., 2018); additional concentrations (1, 10, and 100 µM) were selected for secondary concentration-dependent screening assays (Fig. 2). All drug solutions were prepared by an investigator and coded. A second investigator performed the experiment (e.g., locomotion assay or electrophysiology) blinded to drug identity. Drugs were decoded only after data analysis was completed.

### Locomotion Assay

The acute locomotion assay used here follows protocols established previously (Baraban et al., 2013; Dinday and Baraban, 2015; Griffin et al., 2017; Griffin et al., 2019). At 5 days post fertilization (dpf) offspring from crossing adult *scn1lab^+/^* ^−^ zebrafish were sorted by pigmentation to isolate *scn1lab* ^−^ *^/^* ^−^ larvae and placed individually in one well of a 96-well plate. The plate was positioned in a DanioVision (Noldus) chamber under dark light for a 20-min habituation period at room temperature before obtaining a 10 min “Baseline Recording” epoch. After baseline measurements, embryo media was carefully removed and 75 µl test drug (at a concentration of 10 or 250 µM) or embryo media (internal control, 1% DMSO) were added. Each plate included a set of six internal (vehicle) controls. The plate was returned to the chamber for another 20-min habituation period before beginning a 10 min “Experimental Recording” epoch. Criteria for a positive hit designation were set as follows: (1) a decrease in mean velocity of ≥40% based on an s.d. of 17.83 for internal (vehicle) controls, and (2) a reduction to stage 0 or stage I seizure behavior (defined in Baraban et al., 2005) in the locomotion plot for at least 50% of the test fish. Each test compound classified as a “positive hit” in the locomotion assay was assessed for acute toxicity by direct visualization on a stereomicroscope. Acute toxicity (or mortality) was defined as no visible heartbeat or movement in response to external stimulation in at least 50% of the test fish at 60 min of drug exposure. Compounds identified as successful in the first locomotion screen were re-tested on an independent clutch of larvae using the method described above. Compounds that were successful in two independent locomotion assays, and were not acutely toxic, were re-tested a third time using fresh drug stock sourced from Cayman Chemical.

### Electrophysiology Assay

Zebrafish larvae (5 dpf) were first monitored in the 96-well format locomotion assay as described above. Individual *scn1lab* ^−^ *^/^* ^−^ larvae were then removed, briefly exposed to cold anesthesia and immobilized in 2% agarose by an investigator blinded to the locomotion assay parameter. Local field potential recordings (LFPs) were obtained from midbrain using a single-electrode recording at room temperature, as previously described (Baraban et al., 2005; Baraban, 2013). LFP recordings, 10 min in duration, were obtained using Axoclamp software (Molecular Devices; Sunnyvale, CA) at an acquisition rate of 1 kHz. Abnormal electrographic seizure-like events were analyzed *post hoc* as: (i) brief *interictal-like* events (0.47 ± 0.02 sec duration; n = 52) comprised of spike upward or downward membrane deflections greater than 3x baseline noise level or (ii) long duration, large amplitude *ictal-like* multi or poly-spike events (3.09 ± 1.01 sec duration; n = 21) greater than 5x baseline noise level. Noise level was measured as 0.35 ± 0.03 mV (n = 18). All *scn1lab* mutants exhibit some form of spontaneous electrographic seizure activity and no clustering of activity was observed even with prolonged electrophysiology monitoring (see Hong et al., 2016). Both epileptiform events were counted using “threshold” and/or “template” detection settings in Clampfit (Molecular Devices; Sunnyvale, CA). All embedded larvae were continuously monitored for blood flow and heart rate using an Axiocam digital camera. All experiments were performed on at least two independent clutches of *scn1lab* ^−^ *^/^* ^−^ zebrafish larvae.

### Statistics

Electrophysiological data was examined by one-way analysis of variance with subsequent Dunnett multiple comparisons tests.

## Results

### Behavioral Screening

To identify SC compounds that modify convulsive swim behavior, we performed a blinded *in vivo* primary screen using an automated locomotion tracking protocol. Mutants were placed individually in a single well of a 96-well plate and a baseline locomotion tracking plot was obtained in embryo media. At baseline, all *scn1lab* mutant larvae exhibited spontaneous convulsive behaviors associated with high-velocity movement, as previously reported (Baraban et al., 2013; Dinday and Baraban, 2015; Griffin et al., 2017; Griffin et al., 2019). Mutants were then exposed to test SC compounds at a single screening concentration of 250 µM (n = 6 fish/drug). All compounds remained coded by compound number provided by Cayman Chemical. Mutant swim activity between two consecutive recording epochs in embryo media was tracked on every plate as an internal (vehicle) control. Mean velocity was calculated for each well and the percent change from baseline for all 370 test compounds is plotted in **Figure 1A**; drugs that were acutely toxic are shown in red (also noted in **Supplemental Table I**). On first pass locomotion screening at 250 µM, 27% of SC compounds were identified as positive hits and 39% of SC compounds were identified as acutely toxic (**Figure 1C**). To reduce the percent of compounds being identified as toxic and potential false negatives, a second screen was performed on all 370 compounds at a concentration of 10 µM (n = 6 fish/drug), following the same protocol (**Figure 1B**). On first pass locomotion screening at 10 µM, 25% of SC compounds were identified as acutely toxic and 22% were identified as positive hits (**Figure 1C**). Twenty SC compounds effectively decreased high velocity seizure-like swim behavior at 10 and 250 µM (**Figures 1D, E**).

Next, all 20 SCs compounds which emerged from the two primary screens were decoded and sourced as individual drugs from Cayman Chemical for concentration-response studies using *scn1lab* mutant larvae. SCs were tested at 1, 10, and 100 µM (n = 6 fish/drug/concentration). Five SC compounds were identified as decreasing seizure-like swim behavior observed in *scn1lab* mutant larvae in a concentration-dependent manner (**Figure 2**). Representative locomotion tracking plots are shown in **Figure 2B**. Aggregating data from the first stage of our zebrafish anti-seizure drug screening platform, these five SC compounds were classified as positive hits for reducing seizure-like swim behavior to control levels, and subsequently moved on electrophysiology assays for confirmation of anti-seizure activity.

### Electrophysiology Screening

**Figure 3:**
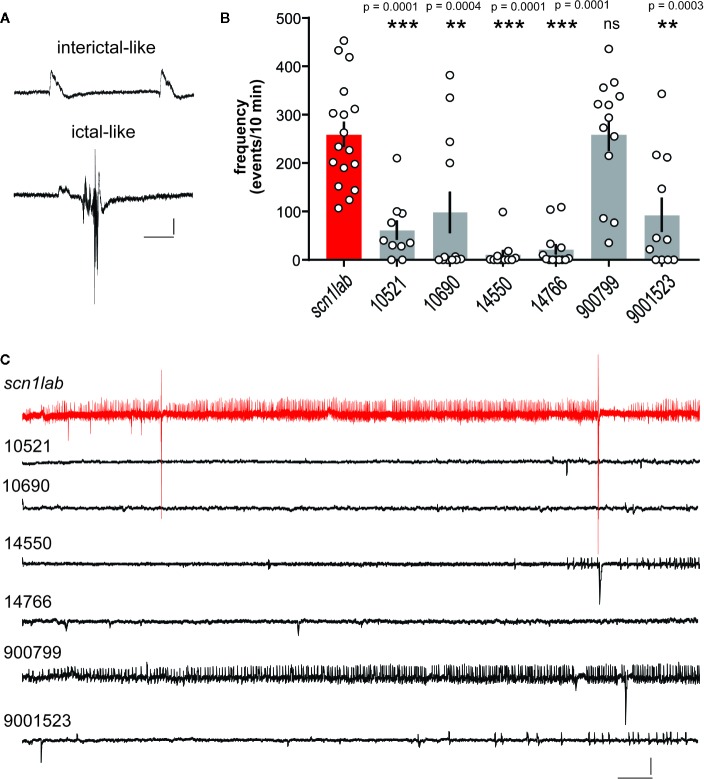
Electrophysiological assay for compounds identified in the locomotion-based screening assay. **(A)** Local field potential (LFP) recordings were obtained with an glass micro-electrode placed under visual guidance in the midbrain of agar-immobilized *scn1lab* larvae that had previously showed reduced seizure-like behavior in the locomotion assay. Representative examples of events classified as interictal- or ictal-like are shown. Scale bars: 1 mV, 0.5 s, **(B)** Bar graphs show the frequency of epileptiform events in a 10 min recording epoch for *scn1lab* larvae exposed to DMSO vehicle (scn1lab mutants; n = 17), JWH 018 N-(5-chloropentyl) analog (compound #10521; n = 10), JWH 018 N-(2-methylbutyl) isomer (compound #10690; n = 12), 5-fluoro PB-22 5-hydroxyisoquinoline isomer (compound #14550; n = 11), 5-fluoro ADBICA (compound #14766; n = 13), JWH 018 adamantyl analog (compound #9000799; n = 13), and AB-FUBINACA 3-fluorobenzyl isomer (compound #90001523; n = 11). Mean ± SEM and individual data points are shown. One-way analysis of variance with Dunnet's multiple comparisons was used to test for significance. **p < 0.001; *** p < 0.0001. **(C)** Representative electrophysiology traces (10 min) are shown for SC compounds 10521, 10690, 14550, 14766, 9000799, and 900015323 compared to an *scn1lab* mutant zebrafish (red). Scale bars: 1 mV, 10 s.

To evaluate whether selected SCs inhibit electrographic seizures, we performed a blinded *in vivo* secondary screen using electrophysiology techniques (Baraban et al., 2005; Baraban, 2013; Baraban et al., 2013; Griffin et al., 2017; Griffin et al., 2019). Mutants were placed in a single well of a 96-well plate and exposed to test SC compounds (#10521, #10690, #14550, #14766, #900799, and #9001523; **Table 1**) at drug concentrations determined above or DMSO (vehicle). Compound #900799 was selected here as a “control” SC that fell below the positive hit threshold described above for the final locomotion-based assay. After the locomotion assay, *scn1lab* mutant larvae were immobilized in agar for LFP recording and *post hoc* analysis of both interictal- (range: 107–453 events/10 min) and ictal-like (range: 1–10 events/10 min) activity (**Figure 3A**). Exposure to compounds #10521, #10690, #14500, #14766, and #9001523 significantly reduced the frequency of these spontaneous epileptiform events (*F*_(6,80)_ = 14.41, ***p < 0.0001 or **p < 0.0003) (**Figure 3B**). Representative 10 min recording epochs for each compound, and an age-matched vehicle-exposed *scn1lab* mutant larvae control are shown in **Figure 3C**. Note the presence of robust interictal- and ictal-like events in *scn1lab* controls and following exposure to compound #900799 but the near complete absence of events with exposure to five different SCs.

**Table 1:** SC compounds identified to have anti-seizure properties in *scn1lab* mutant zebrafish larvae.

Compound no.	Compound name	Description
**10521**	JWH 018 N-(5-chloropentyl) analog	JWH 018 is a SC that potently activates the central cannabinoid (CB1) and peripheral cannabinoid (CB2) receptors (Ki = 9.0 and 2.94 nM, respectively). JWH 018 N-(5-chloropentyl) analog differs structurally from JWH 018 by having chlorine added to the five position of the pentyl chain (Aung et al., 2000; Lindigkeit et al., 2009; Uchimaya et al., 2009).
**10690**	JWH 018 N-(2-methylbutyl) isomer	JWH 018 2-methylbutyl homolog is an analog of JWH 018, a mildly selective agonist of the central cannabinoid receptor (Ki = 8.9 nM) derived from the aminoalkylindole WIN 55,212-2 (Aung et al., 2000).
**14550**	5-fluoro PB-22 5-hydroxyisoquinoline isomer	5-Fluoro PB-22 is an analog of 5-fluoropentyl JWH-type cannabimimetics. 5-Fluoro PB-22 5-hydroxyisoquinoline isomer differs from 5-fluoro PB-22 by having the quinoline group replaced with an isoquinoline group attached at its five position (Uchimaya et al., 2009).
**14766**	5-fluoro ADBICA	This compound is a derivative of ADBICA featuring a fluorine atom added to the terminal carbon of the pentyl group (Uchiyama et al., 2012).
**9000799**	JWH 018 adamantyl analog	JWH 018 adamantyl analog is a mildly selective agonist of the peripheral cannabinoid receptor, where the naphthalene ring is substituted with an adamantyl group (Lu et al., 2005; Sobolevsky et al., 2010).
**9001523**	AB-FUBINACA 3-fluorobenzyl isomer	AB-FUBINACA is an indazole-based SC that potently binds the central CB1 receptor (Ki = 0.9 nM) (Uchiyama et al., 2012).

SC, synthetic cannabinoid.

## Discussion

Here we describe the first large-scale phenotype-based screen of a SC library using a zebrafish model of Dravet syndrome. Our approach to screening compounds for anti-seizure activity in *scn1lab* mutant zebrafish builds on earlier model validation, established acute drug exposure protocols, and a database now exceeding 3,200 compounds (Baraban et al., 2013; Kumar et al., 2016; Griffin et al., 2016; Hong et al., 2016; Grone et al., 2017; Griffin et al., 2017; Griffin et al., 2018). Using a two-stage screening strategy in *scn1lab* mutant zebrafish, and anti-seizure criteria incorporating electrophysiology read-outs, we identified five SCs. SCs add to a growing list of drugs successfully identified in *scn1lab* zebrafish that already include “standard-of-care” AEDs (e.g., valproate, stiripentol, benzodiazepines, bromides) and experimental drugs (e.g., fenfluramine, lorcaserin, trazodone, clemizole) (Baraban et al., 2013; Dinday and Baraban, 2015; Griffin et al., 2017; Griffin et al., 2019).

SCs, first developed in the 1940s, represent a variety of compounds engineered to bind cannabinoid receptors with high affinity. Many SCs bind G-protein coupled CB1 receptors located at the presynaptic terminal and are also thought to interact with voltage-gated potassium channels, voltage-gated sodium channels, N-and P/Q-type-calcium channels, and an orphan G-protein coupled receptor (GPR55) (for review see Rosenberg et al., 2017; Smolyakova et al., 2020). Whether one, or several, of these potential sites of action are responsible for anti-seizure effects noted with SCs remains a controversial area of investigation as, cannabinoid-receptor dependent, and independent, pro- or anticonvulsant effects have been reported. Complex differences in cannabinoid receptor expression on excitatory versus inhibitory neurons in the brain, as well as the wide variety of preclinical models in which cannabinoids anti-seizure or anti-epileptogenic effect are studied, has probably contributed to this controversy. For example, *in vivo* studies using intracerebroventricular administration of a CB1-receptor agonist, arachidonyl-2-chloroethylamide (ACEA), significantly decreased the frequency of penicillin-induced epileptiform activity in rats (Kozan et al., 2009). CMYL-4CN-BINACA, another CB1 receptor agonist, elicited pro-convulsant effects in mice (Kevin et al., 2019) and a SC (AM2201) induced epileptiform activity and convulsive behaviors in mice that could be blocked by the selective CB1 receptor antagonist AM251 (Funada and Takebayashi-Ohsawa, 2018). Huizenga et al. (2017) reported that nonselective CB1/2 and selective CB1 agonists suppress activity in neonatal seizure models (though these findings were not replicated with GPR55 agonists). WIN55,212, a synthetic CB1R agonist, elicited anti-convulsant effects in several acute seizure models of acute seizure (Wallace et al., 2001; Naderi et al., 2008) whereas oral administration of SR141716A (rimonabant), a CB1R specific antagonist, increased seizure susceptibility and duration, suggesting a pro-convulsant effect (Vinogradova et al., 2011). Unfortunately, the majority of these preclinical studies utilized adult rodent models, were not focused on pediatric epilepsy conditions where modulation of endocannabinoid signaling may be most therapeutic, and only compared one (or a few) compound(s) in a single study. In contrast, our results using several hundred compounds in a zebrafish model of Dravet syndrome could represent a new strategy to decipher pro- versus anti-convulsant activities.

**Figure 4:**
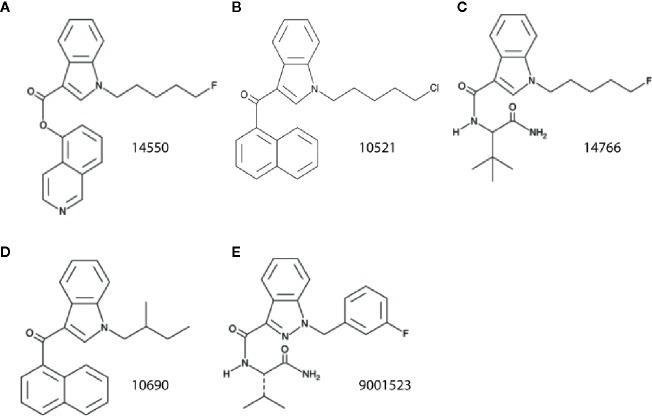
Structural comparison of SC identified to reduce spontaneous seizures in the *scn1lab* Dravet syndrome zebrafish mutant. **(A)** 14550; 5-fluoro PB-22 5-hydroxyisoquinoline isomer, **(B)** 10521; JWH 018 N-(5-chloropentyl) analog, **(C)** 14766; 5-fluoro ADBICA, **(D)** 10690; JWH 018 N-(2-methylbutyl) isomer, and **(E)** 9001523; AB-FUBINACA 3-fluorobenzyl isomer.

Zebrafish possess orthologs for 82% of human disease-associated genes (Howe et al., 2013). Orthologous zebrafish proteins are often similar to human within their functional domains. For example, between 66% and 75% of amino acids in the zebrafish Cb1 receptor are similar to those in human CB1 (Lam et al., 2006); comparison of the zebrafish Cb2 (a/b) receptor revealed a 39% amino acid similarity with human CB2 (Rodriguez-Martin et al., 2007). Both receptors are expressed in the zebrafish central nervous system. These properties combined with ease of large-scale pharmacological screening make larval zebrafish a good model system for investigating drug targets. A recent study (Samarut et al., 2019), limited only to locomotion assay read-outs in larval zebrafish, reported a synergistic effect of Δ-9-tetrahydrocannabinol (THC) and CBD on hyperactive behaviors. Unfortunately, these studies failed to include electrophysiology measures and were restricted to these two compounds. Here we initially screened 370 different SCs in a locomotion assay at two different drug concentrations, identifying 20 compounds. With repeated biological replicates, newly sourced compound re-tests and sensitive secondary electrophysiology assays, we narrowed this initial list to five SCs classified as effective in suppressing spontaneous seizures in *scn1lab* mutants. This two-stage approach further validates the selectivity of our screening strategy as only 1.3% of all SCs tested were identified as true anti-seizure hits. A caveat of this strategy is that pharmacokinetic data for how SCs are absorbed, distributed or metabolized in larval zebrafish is not available which would suggest an error in false negative designations. Although we also recognize that acute SC exposure may alter non-seizure larval behavior in wild-type larvae (Achenbach et al., 2018; Ellis et al., 2018; Luchtenburg et al., 2019), our studies are focused on Dravet syndrome zebrafish as the overall purpose of this research was to identify anti-seizure compounds. Interestingly, as several of these SCs are classified as indole-derivatives these data also suggest a potential target not recognized in previous preclinical studies (**Figure 4**). Indole-derived cannabinoids have strong binding affinity for 5HT_2B_ receptors (Wiley et al., 2016). This serotonin receptor subtype was recently identified by our group as potentially mediating anti-seizure effects of clemizole (Griffin et al., 2019). Taken together, a convergence of evidence now exists to suggest that activation of a serotonin 2B receptor is a potential target for Dravet syndrome therapy.

Although additional preclinical studies investigating SCs identified here (both in wild-type and epileptic zebrafish) are warranted, a note of caution needs to be extended. First and foremost, SCs are not intended “for human or veterinary use” (https://www.caymanchem.com/product/9002891). Second, substantial evidence of serious adverse effects have been reported for some of these compounds. These include, and may not be limited to, panic attacks, memory distortions, paranoia, psychotic reactions, disorganized behavior, and suicidal thoughts (Fattore, 2016; Tait et al., 2016). Reports of acute ischemic stroke (Freeman et al., 2013; Takematsu et al., 2014), seizures (Schneir and Baumbacher, 2012; Schwartz et al., 2015), and sudden death (Behonick et al., 2014; Shanks et al., 2016; Shanks and Behonick, 2016) with SCs are not uncommon. In particular, indole- and indazole-based SCs are associated with clinical signs of reduced consciousness, paranoia and seizures in humans (Hill et al., 2018). This information is valuable for investigators and clinician-scientists interested in the potential benefits of cannabinoid receptor agonists for intractable seizure disorders.

## Data Availability Statement

The datasets generated for this study are available on request to the corresponding author.

## Ethics Statement

The animal study was reviewed and approved by Office of Ethics and Compliance branch of the Institutional Animal Care and Use Committee at the University of California, San Francisco.

## Author Contributions

Locomotion-based screening assays were performed by MA, KH, and AG. Electrophysiology experiments were performed by SB. The design, conceptualization, interpretation of data, data analysis, and writing of the manuscript was done by AG and SB. All authors contributed to the editing process.

## Funding

This work was supported from NINDS R01 grants no. NS096976 and NS103139 (SB).

## Conflict of Interest

SB is a co-Founder and Scientific Advisor for EpyGenix Therapeutics. SB is on the Scientific Advisory Board of ZeClinics.

The remaining authors declare that the research was conducted in the absence of any commercial or financial relationships that could be construed as a potential conflict of interest.
